# A High-Resolution Stereo-Seq Spatial Transcriptomic Resource for Adult Holstein Cattle Liver

**DOI:** 10.3390/ijms27177844

**Published:** 2026-09-02

**Authors:** Shamima Akter, Congjun Li, Nayan Bhowmik, Liu Yang, Li Ma, Curtis P. Van Tassell, Ransom L. Baldwin VI, George E. Liu

**Affiliations:** 1 Animal Genomics and Improvement Laboratory, Beltsville Agricultural Research Center, Agricultural Research Service, United States Department of Agriculture, Beltsville, MD 20705, USA; 2 Department of Animal and Avian Sciences, University of Maryland, College Park, MD 20742, USA

**Keywords:** Stereo-seq, spatial transcriptomics, bovine liver, Holstein cattle, spatial gene expression, livestock genomics

## Abstract

The bovine liver is a highly compartmentalized organ that plays essential roles in continuous gluconeogenesis and nitrogen recycling; however, its spatial molecular architecture has remained largely uncharacterized due to the limitations of traditional bulk and single-cell approaches. To address this gap, Spatial Enhanced Resolution Omics-sequencing (Stereo-seq) was utilized to generate a subcellular-resolution (500 nm) transcriptomic map of an adult Holstein cattle liver, and a refined reference-guided workflow was implemented to overcome standard annotation limitations in livestock. Raw sequencing data were processed using the Stereo-seq Analysis Workflow and analyzed with Stereopy, Seurat, SingleR, and reference-guided workflows. Spatial aggregation was evaluated at Bin20, Bin50, Bin100, Bin150, and Bin200. Increasing bin size increased molecular identifier counts and detected-gene complexity while progressively reducing spatial granularity. Bin50, corresponding to 50 × 50 DNA nanoballs and an approximate nominal footprint of 25 × 25 µm, was therefore selected as a practical intermediate aggregation level for the primary analyses. Quality-control assessment, Leiden clustering, UMAP visualization, reference-based cell-type annotation, cluster-marker analysis, and spatial mapping of canonical hepatic genes demonstrated preservation of biologically interpretable liver transcriptional organization. Raw sequencing data processed spatial matrices, annotated objects, and analysis code are publicly available to support reanalysis and computational benchmarking. In summary, we present a Stereo-seq spatial transcriptomic resource generated from liver tissue of an adult Holstein cow. This initial resource provides a valuable foundation for future studies of bovine liver biology, comparative genomics, and the spatial basis of livestock health and production traits.

## 1. Introduction

The liver performs diverse metabolic, detoxification, and synthetic functions that are spatially organized along the lobular axis [1,2,3]. Cattle absorb relatively little glucose directly from the gastrointestinal tract and therefore rely heavily on continuous hepatic gluconeogenesis. This process is fueled mainly by rumen-derived volatile fatty acids (VFAs), particularly propionate [4,5,6,7]. Transcriptomic studies have substantially improved our understanding of liver function. Traditional bulk RNA sequencing has been widely used to profile liver gene expression in livestock and model organisms; however, it averages signals across heterogeneous cell populations and anatomical regions (RNA-seq), masking these spatial patterns, and typically loses positional context because of tissue dissociation [8]. Spatial transcriptomic technologies overcome these limitations by preserving tissue architecture while profiling transcriptomes [9,10].

Stereo-seq is a high-resolution spatial transcriptomics technology that combines dense DNA nanoball (DNB) arrays and spatial barcodes to capture mRNA directly from tissue sections at near single-cell or subcellular resolution across large tissue areas [11]. This approach has been applied to developmental biology, tissue atlases, organ regeneration, cancer, and comparative spatial transcriptomics in several species such as humans and mice [12,13,14,15,16]. Moreover, Stereo-seq has been used to investigate placental development in dairy cattle [17], while Visium-based spatial transcriptomics has been applied to broiler skeletal muscle and porcine reproductive tissues [PMID: 38342952, PMID: 40875599]. High-resolution Stereo-seq datasets have also been generated from avian tissues, including chicken lung tissues [PMID: 40177910]. In the liver, recent comparative studies have included humans, cattle, and pigs and have provided important information on conserved mammalian hepatic organization [PMID: 41986723]. The present study complements these efforts by providing an adult Holstein liver Stereo-seq resource and data generator. Here, we describe a Stereo-seq dataset generated from an adult Holstein liver and provide a reproducible computational workflow for quality control, spatial aggregation, unsupervised clustering, reference-guided annotation, marker-gene evaluation, and spatial visualization. To date, Stereo-seq technology has provided critical biological insights into the spatial architecture of human and mouse livers. However, this technology has not yet been applied to livestock. Here, we present the first Stereo-seq–based spatial transcriptomic dataset for the cattle (Holstein) liver, addressing a major gap in ruminant functional genomics. Raw sequencing reads were processed with the Stereo-seq Analysis Workflow (SAW), and downstream analyses were performed using Stereopy, Seurat, SingleR, and Scanpy-based workflows. Spatial gene-expression matrices, quality-control summaries, Leiden clusters, UMAP embeddings, reference-based cell-type annotations, marker-gene visualizations, and reusable annotated data objects were generated. The study is intentionally focused on technical characterization and reuse of the dataset, rather than inference of biological differences. We additionally evaluate Bin20, Bin50, Bin100, Bin150, and Bin200 to establish a dataset-specific rationale for the selected primary resolution. Without adding additional experimental results, this manuscript focuses on improved presentation of the dataset, technical validation, spatial annotation, and reuse potential. The resulting resource provides a foundation for bovine liver cell-type mapping, hepatic zonation studies, livestock trait research, and computational benchmarking of spatial transcriptomic methods. In addition to raw and processed files, analysis scripts and recommended pipelines were provided to reproduce core QC, clustering, and visualization steps.

## 2. Results

### 2.1. Generation of a High-Resolution Spatial Transcriptomic Dataset from Adult Cattle Liver

**Figure 1:**
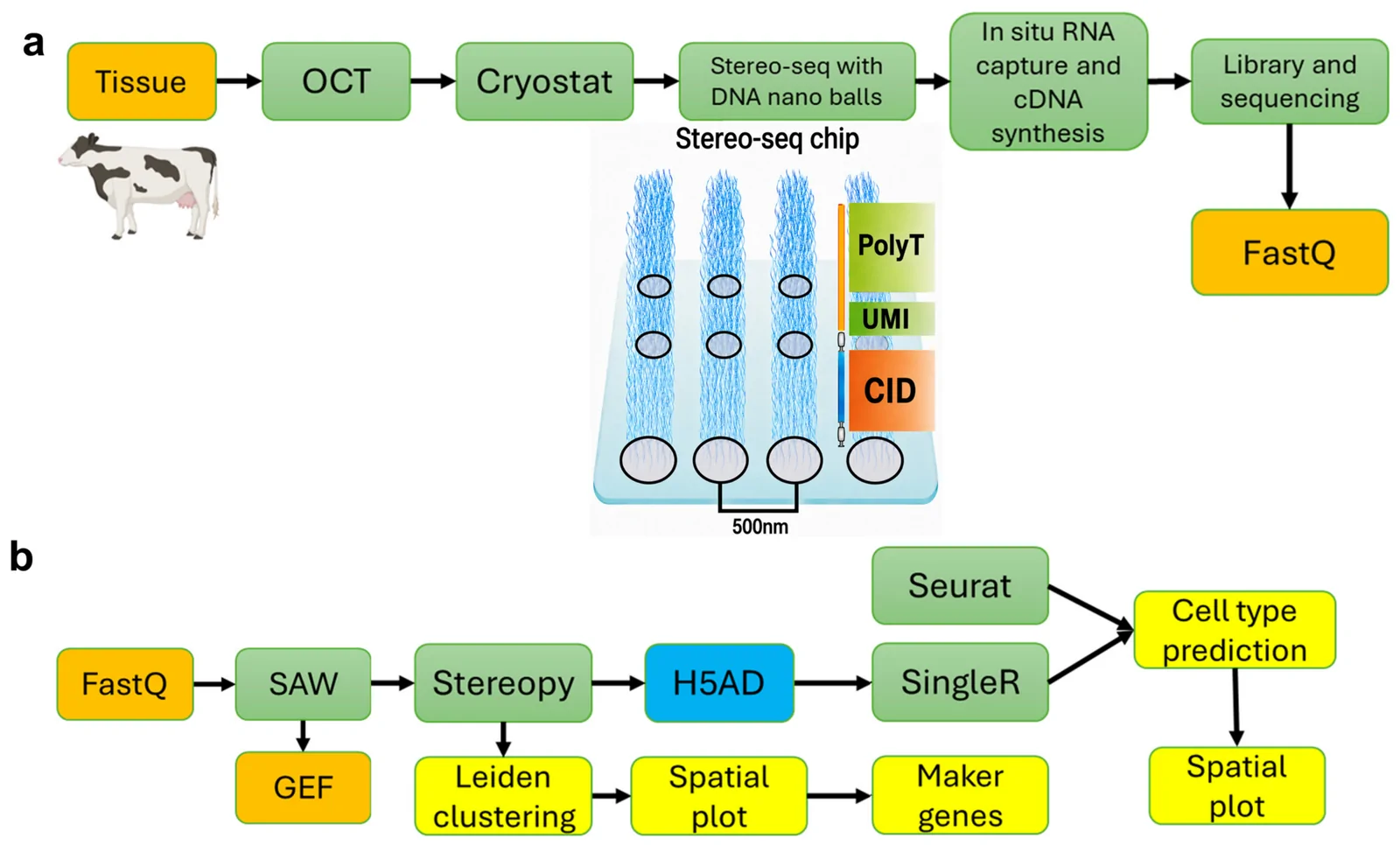
Overview of sample preparation and Stereo-seq workflow. (**a**) Adult Holstein cattle liver tissue was collected, embedded in OCT, cryosectioned, mounted onto Stereo-seq DNA nanoball chips, processed by on-chip RNA capture and cDNA synthesis, and sequenced. (**b**) Raw FASTQ files were processed using SAW v7.0.0 to generate GEF and HDF5-compatible outputs. Downstream analyses were performed using Stereopy, Seurat, and SingleR for quality control, Leiden clustering, marker-gene detection, spatial visualization, and cell-type prediction.

The experimental and computational workflow is summarized in Figure 1. Processed spatial expression outputs included tissue.gef and tissue.raw.gef files, which were subsequently used for quality control, bin-level aggregation, clustering, annotation, and visualization.

For downstream analysis, the Stereo-seq matrix was aggregated into Bin50 spatial organization and converted into h5ad format for compatibility with AnnData-based workflows. Stereopy was used for preprocessing and spatial visualization, while Seurat and SingleR were used for clustering, marker-gene analysis, and reference-based cell-type annotation. The processed dataset, therefore, supports both Stereo-seq-specific and broadly used single-cell/spatial transcriptomic analysis environments.

### 2.2. Quality-Control Assessment of Spatial Gene-Expression Data

Quality-control analysis was performed at multiple stages to evaluate the quality and usability of the spatial transcriptomic dataset. At the sequencing and primary processing levels, reads were filtered to remove low-quality reads, reads containing ambiguous bases in cellular or molecular identifier regions, and reads with low average base quality. After alignment and UMI collapsing, bin-level quality metrics were calculated from the processed expression matrix.

**Figure 2:**
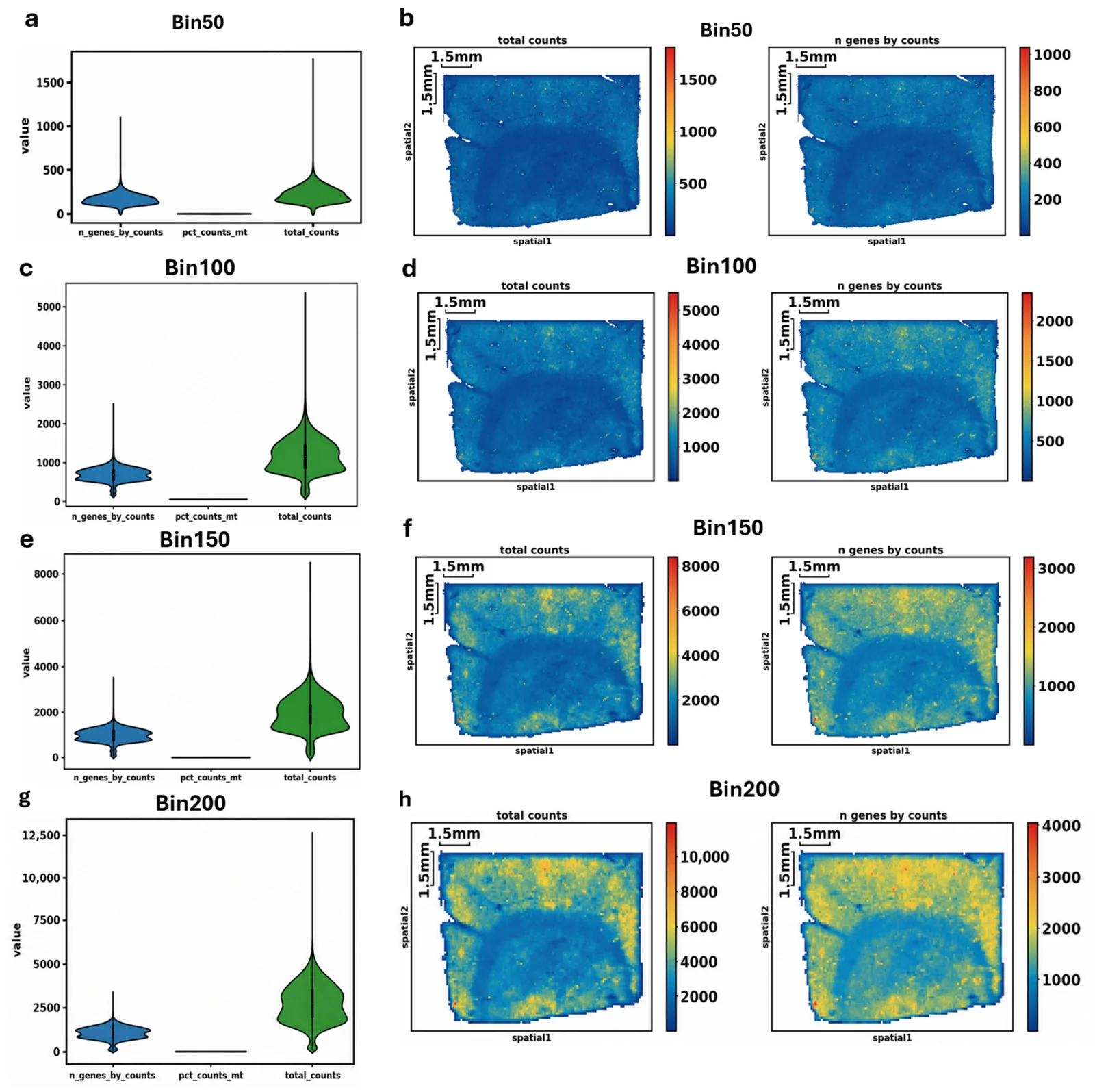
Quality-control assessment across Stereo-seq aggregation levels. (**a**,**b**) Bin50; (**c**,**d**) Bin100; (**e**,**f**) Bin150; and (**g**,**h**) Bin200. For each aggregation level, Violin plots show the distributions of the number of detected genes per spatial bin (n_genes_by_counts), the percentage of counts assigned to mitochondrial genes (pct_counts_mt), and total molecular counts per spatial bin (total_counts). The width of each violin represents the relative density of spatial observations at a given value. Detected-gene and total-count distributions show a predominant population of bins with moderate transcriptomic complexity together with smaller upper-tail populations exhibiting increased molecular recovery. Spatial maps show the tissue distribution of molecular and detected gene counts. Larger spatial units contain progressively greater transcriptomic complexity, whereas finer aggregation retains greater spatial granularity.

The main quality-control metrics included total UMI counts, detected gene counts, and mitochondrial transcript proportion per spatial bin. Violin distributions and spatial maps summarize transcriptomic complexity across Bin50, Bin100, Bin150, and Bin200. Panels show detected-gene counts, total molecular counts, and mitochondrial-transcript proportions where applicable, together with spatial projections of total counts and detected genes over the liver tissue section (Figure 2). Increasing spatial aggregation produced an expected increase in per-bin molecular and gene recovery while preserving broad tissue coverage. Mitochondrial transcripts were removed during downstream processing and were therefore present at very low levels in the processed matrices used for bin-level quality-control analysis. Figure 2 therefore focuses primarily on spatial-bin quality based on detected gene and molecular counts and helps identify low-complexity or extreme observations.

The processed dataset supported downstream dimensionality reduction, Leiden clustering, marker-gene detection, and spatial visualization, indicating that the dataset is suitable for spatial transcriptomic analysis of adult cattle liver.

### 2.3. Spatial Clustering Identifies Transcriptional Domains in Adult Cattle Liver

**Figure 3:**
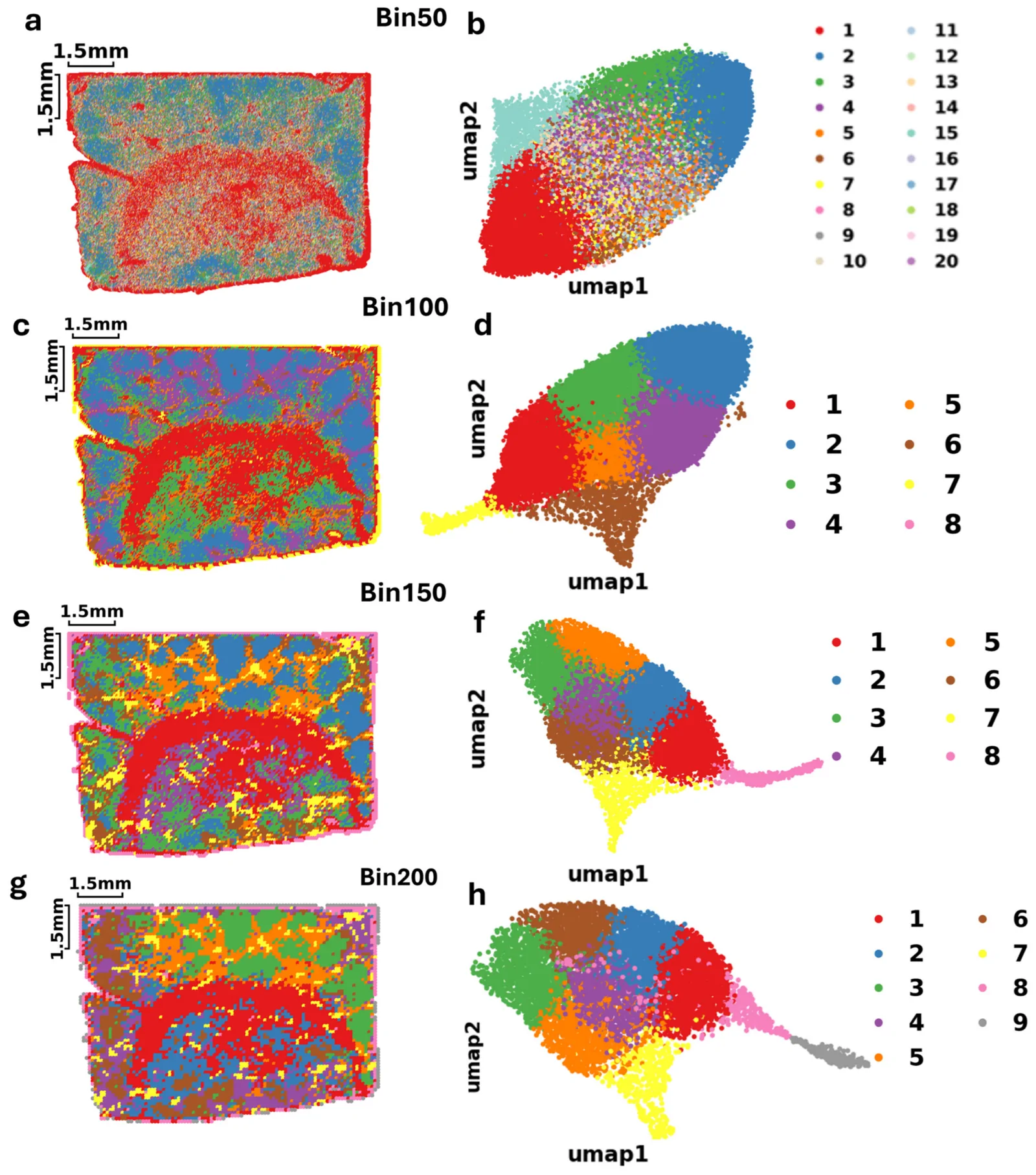
Effect of spatial aggregation on Leiden clustering and UMAP structure. (**a**,**b**) Bin50 spatial cluster map and UMAP; (**c**,**d**) Bin100; (**e**,**f**) Bin150; and (**g**,**h**) Bin200. Resolution-dependent changes in cluster granularity are evident as increasingly larger DNB regions are aggregated, while major transcriptional organizations remain visually apparent across aggregation levels.

To identify transcriptionally distinct spatial domains, normalization, dimensionality reduction, neighborhood graph construction, and Leiden clustering were performed on the Bin50 to Bin200 expression matrices. Paired spatial Leiden-cluster maps and UMAP representations are shown for Bin50, Bin100, Bin150, and Bin200, as we decided to exclude Bin20 because it resulted in clusters that were less biologically interpretable. The finer Bin50 aggregation resolves greater transcriptional granularity, whereas progressively larger spatial units merge expression information over broader regions and yield coarser domain structures. Nevertheless, organized transcriptional structure remains qualitatively evident in both spatial coordinates and low-dimensional representations across the examined aggregation levels. This comparison is intended as a qualitative resolution-sensitivity assessment; formal cross-resolution cluster-equivalence statistics were not calculated. Leiden clustering identified 20, 8, 8, and 9 spatial clusters across the cattle liver tissue section at Bin50, Bin100, Bin150, and Bin200, respectively (Figure 3). When projected back onto tissue coordinates, these clusters showed spatial organization across the liver section rather than random distributions, suggesting that the transcriptomic domains reflected underlying tissue structure and spatial heterogeneity.

UMAP visualization further separated transcriptionally distinct bin populations (Figure 3). Spatial clustering is particularly useful in liver because hepatic cell types and hepatocyte functional states are organized across anatomical and microenvironmental gradients. The spatial organization observed in the Leiden clusters provided an initial framework for annotating major liver cell populations and examining canonical hepatic marker-gene localization, which can be further utilized for characterizing the lobular structure of cattle liver.

### 2.4. Reference-Based Annotation Recovers Major Liver Cell Populations

**Figure 4:**
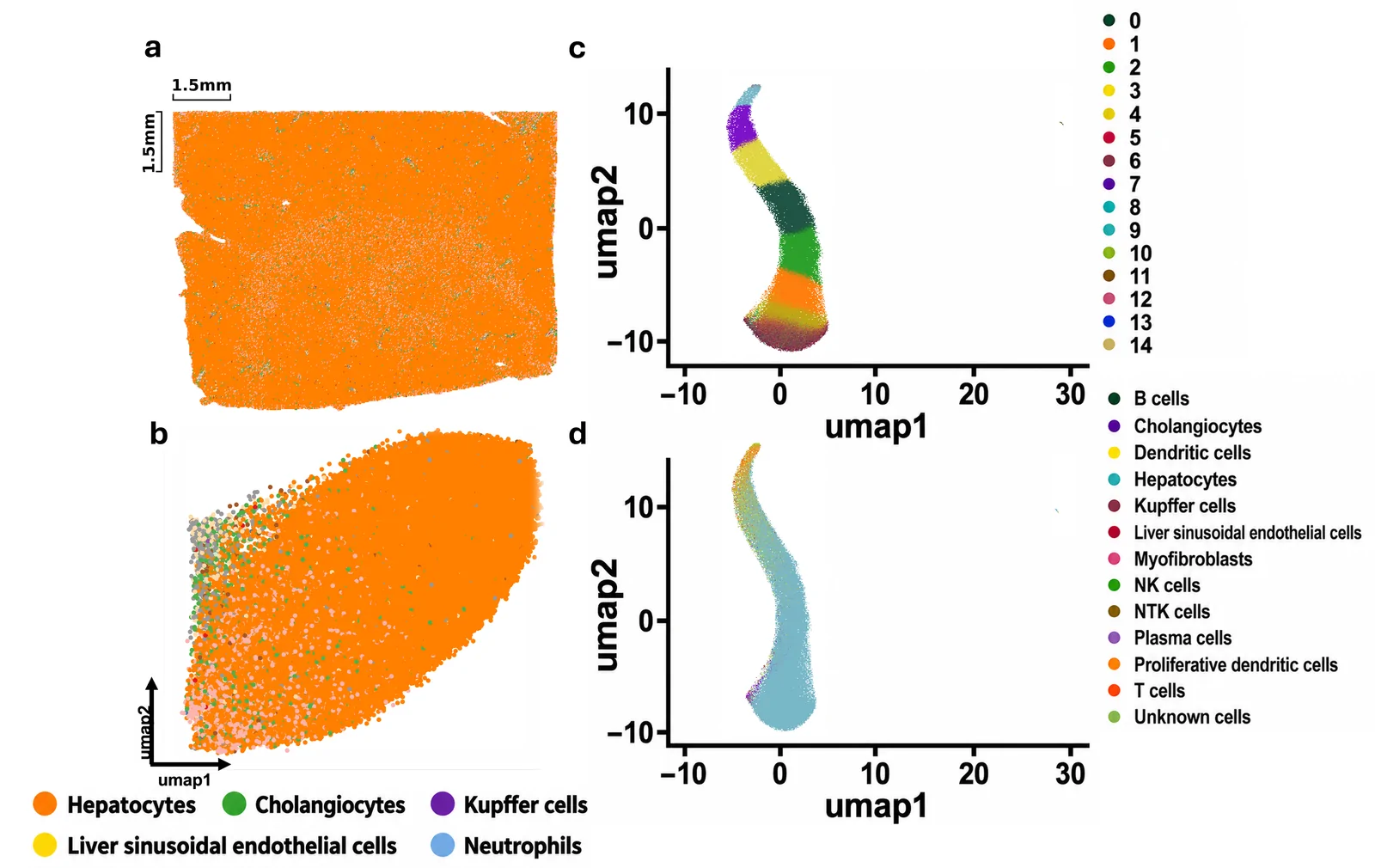
Reference-guided annotation of major cell-type-associated spatial populations in adult Holstein liver. (**a**) Spatial visualization of SingleR cell type clusters across the liver tissue section. (**b**) UMAP projection of SingleR cell type clusters. (**c**) Seurat-based UMAP visualization of spatial clusters. (**d**) UMAP projection of Seurat-annotated cell types. Seurat-based cell-type annotations show major liver cell populations, including hepatocytes, cholangiocytes, Kupffer cells, liver sinusoidal endothelial cells, and immune-cell populations.

To assign biological identities to these spatial domains, a reference-guided annotation framework was implemented. SingleR was first utilized to map the spatial bins to a bovine-specific scRNA-seq reference, revealing the precise anatomical distribution of cell types (Figure 4a) and their corresponding identities in UMAP space (Figure 4b). As expected, the resulting spatial map was dominated by hepatocyte-associated bins, mirroring the known cellular composition of the mammalian liver. Spatial and UMAP representations show reference-based cell-type assignments generated from the primary Bin50 dataset using complementary annotation workflows. Hepatocyte-associated bins dominate the tissue section, with additional assignments corresponding to cholangiocytes, Kupffer cells, liver sinusoidal endothelial cells, neutrophils, and other immune or stromal populations. Concordance among reference prediction, unsupervised clustering and spatial localization was used to support major cell-type interpretations (Figure 4c). Broader reference-based labels, including B cells, T cells, NK/NTK-associated populations, dendritic-cell-associated bins, plasma cells, myofibroblast-associated bins, and unclassified/unknown cells, were also obtained with Seurat reference-based annotation algorithms (Figure 4d). Because Bin50 represents aggregated capture positions rather than explicit single-cell segmentation, labels should be interpreted as cell-type-associated spatial bins.

To further validate these cell-type annotations, we performed differential expression analysis using Seurat’s FindAllMarkers function for the major annotated cell populations, including hepatocytes, cholangiocytes, and Kupffer cells. Up to 20 of these differentially expressed genes (DEGs), provided in Supplementary Table S1, confirmed expected cell-type-specific transcriptional programs. Hepatocyte DEGs were enriched for genes associated with liver metabolism, plasma protein synthesis, lipid transport, coagulation, and acute-phase response. Cholangiocyte DEGs supported biliary epithelial identity, whereas Kupffer cell DEGs reflected macrophage-associated immune and phagocytic functions. Therefore, the combined evidence from Stereo-py cluster markers, Seurat-based cell-type DEGs, spatial localization, and reference-guided annotation demonstrates the reliability of the processed Stereo-seq dataset and supports its value as a reusable resource for bovine liver spatial transcriptomics.

### 2.5. Highly Expressed Genes Reflect Dominant Hepatic Transcriptional Programs

**Figure 5:**
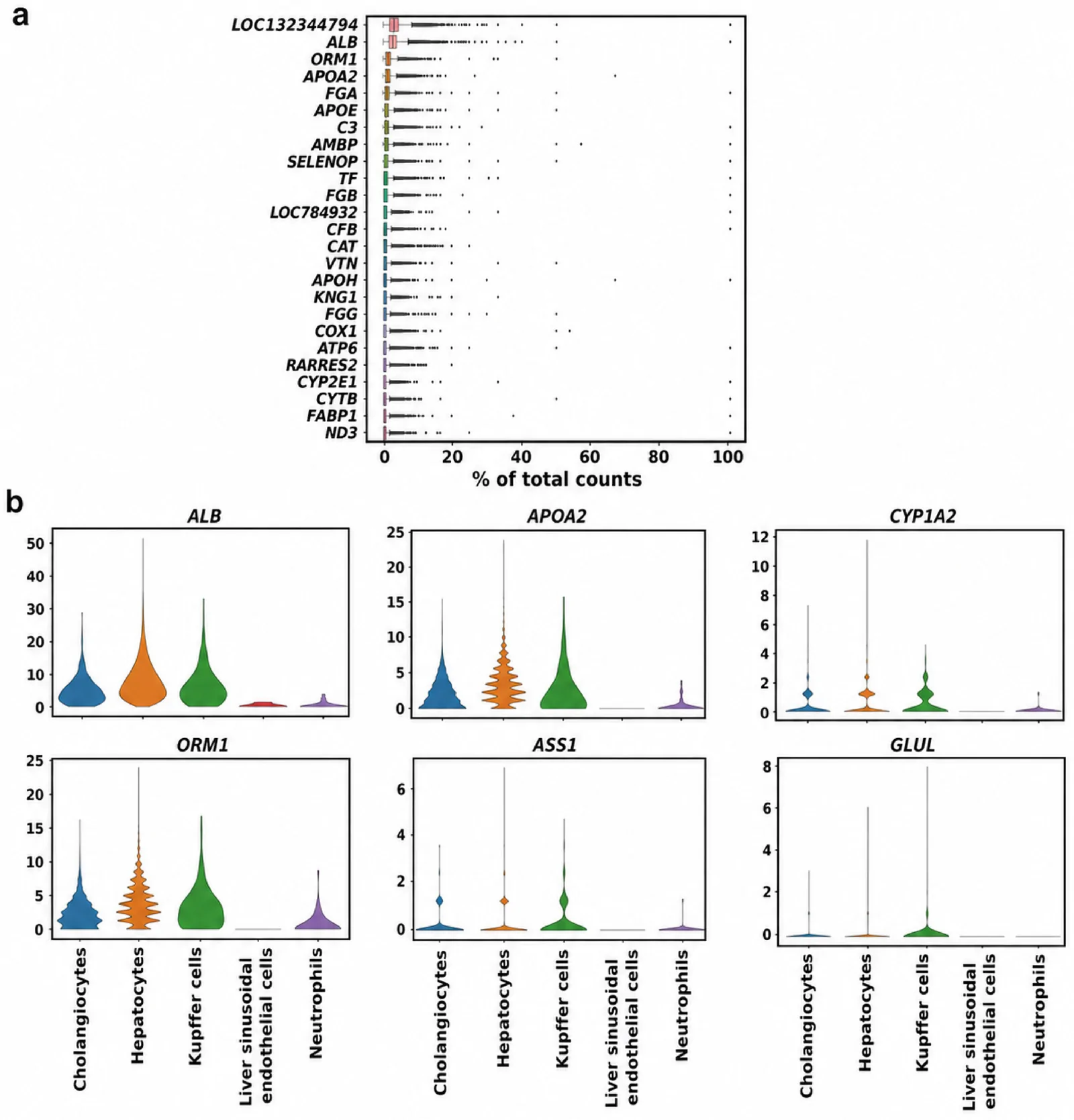
Highly expressed hepatic genes and their distribution across annotated spatial populations. (**a**) Top 25 highly expressed genes in the cattle liver Stereo-seq dataset. (**b**) Violin plots showing expression of selected hepatic genes, including *ALB*, *APOA2*, *CYP1A2*, *ORM1*, *GLUL*, and *ASS1*, across annotated cell types.

To evaluate major transcriptional signals in the adult cattle liver dataset, the top 25 highly expressed genes across the processed spatial transcriptomic matrix were identified. Highly expressed genes included canonical liver-associated and hepatocyte-enriched transcripts such as *ALB* (Albumin), *APOA2* (Apolipoprotein A2), *CYP1A2* (Cytochrome P450 family 1 subfamily A member 2), *ORM1* (Orosomucoid 1), *GLUL* (Glutamate-ammonia ligase), and *ASS1* (Argininosuccinate synthase 1), and other genes associated with plasma protein production, lipid metabolism, complement activity, coagulation, transport, and hepatic metabolic function (Figure 5a).

Because hepatocytes represent the dominant parenchymal population in liver tissue, many of the most abundant transcripts were strongly associated with hepatocyte-like bins. Violin plots of selected major genes across annotated cell types showed high expression of *ALB*, *APOA2*, and *ORM1* in hepatocyte-associated populations, consistent with their known roles in liver function (Figure 5b). *GLUL* showed a more restricted expression pattern compared with broadly expressed hepatocyte markers, consistent with its known association with hepatic zonation.

The expression patterns of these genes support the validity of the cell-type annotations and demonstrate that the dataset captures biologically meaningful hepatic transcriptional programs. These results further indicate that the processed spatial matrices can be used to characterize both broad liver cell identities and spatially restricted expression of hepatic marker genes. Together, these gene-level patterns provide additional support for hepatic tissue identity and highlight differences between broadly expressed and spatially restricted transcriptional programs.

### 2.6. Spatial Expression of Canonical Liver Markers Supports Hepatic Tissue Organization

**Figure 6:**
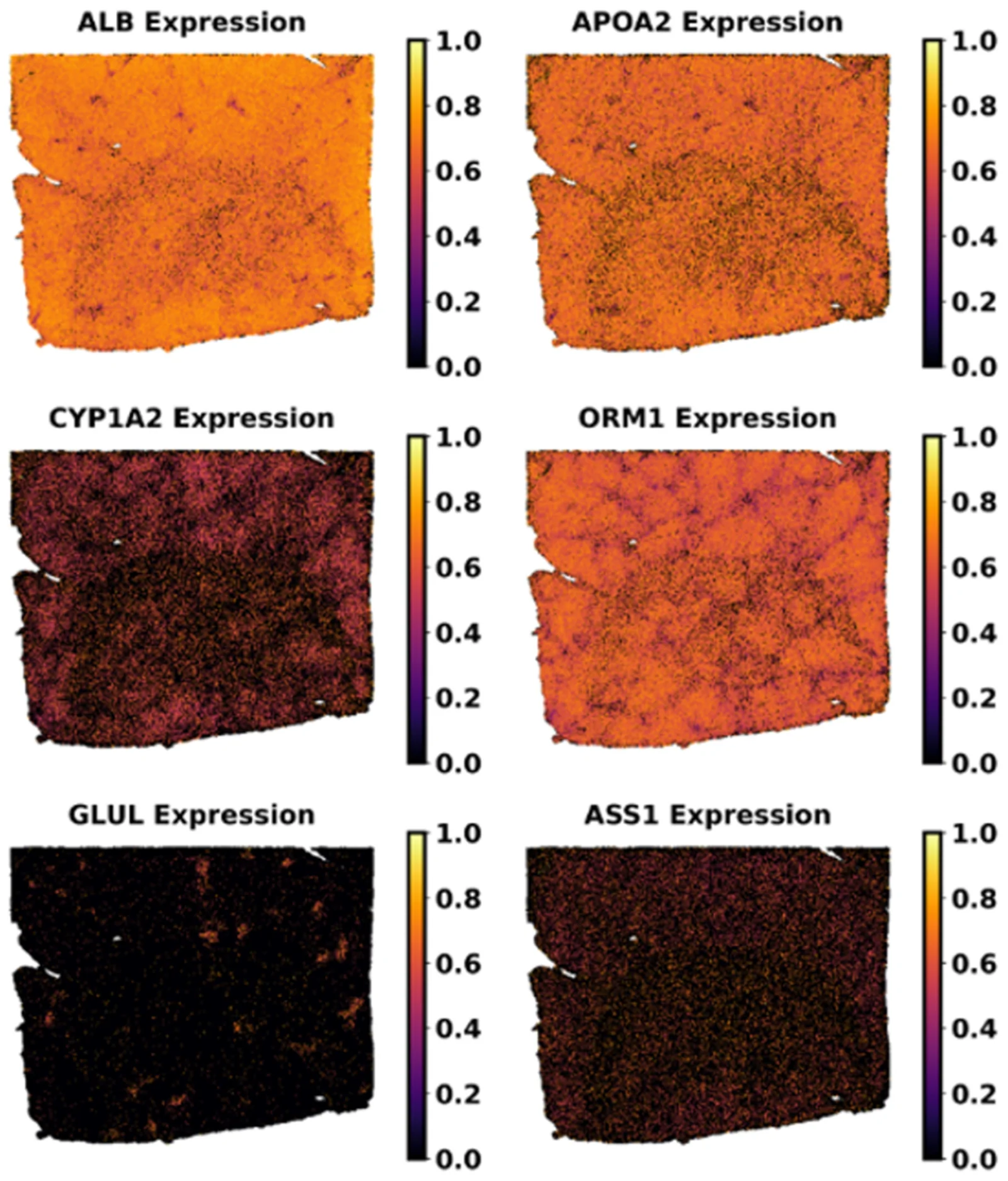
Spatial distribution of canonical hepatic marker genes in adult cattle liver. Spatial expression maps for *ALB*, *APOA2*, *CYP1A2*, *ORM1*, *GLUL*, and *ASS1* in adult cattle liver tissue. These markers support hepatocyte identity, liver-specific transcriptional activity, and spatially variable hepatic gene-expression patterns. Color scales represent the expression levels.

To further validate the spatial transcriptomic dataset, the tissue-level localization of canonical hepatic marker genes was examined. Spatial expression maps were generated for *ALB*, *APOA2*, *CYP1A2*, *ORM1*, *GLUL*, and *ASS1* (Figure 6). *ALB* was broadly expressed across the liver section, consistent with hepatocyte abundance and the central role of albumin production in liver function. *ORM1* also showed spatial expression across the tissue, reflecting hepatic acute-phase and plasma protein transcriptional activity. *GLUL* and *ASS1* expression appeared more spatially restricted than *ALB*, consistent with its established association with pericentral hepatocyte populations in mammalian liver. *CYP1A2* also showed spatially variable expression, supporting the ability of the dataset to capture genes involved in xenobiotic metabolism and zonated hepatic function. Together, these marker-gene maps provide an internal biological validation of the tissue identity, annotation quality, and spatial resolution of the dataset.

## 3. Discussion

This study provides an adult Holstein liver Stereo-seq resource together with a reproducible workflow for spatial aggregation, quality control, clustering, cell-type annotation, and gene-level visualization. Its principal contribution is the generation and technical characterization of a cattle liver spatial-transcriptomic dataset that can be reanalyzed using Stereo-seq-specific and general spatial transcriptomic computational approaches.

The technical validation demonstrated consistent transcript recovery and spatial coverage across the tissue section. Increasing bin size produced the expected increase in molecular counts and detected-gene complexity, while reducing spatial granularity (Figure 2 and Figures S1–S4). Sequencing saturation analysis (Figure S3) assessed the relationship between sequencing depth and molecular recovery, while tissue statistics and Tissue Cut summaries (Tables S1 and S2) characterized sequencing performance, tissue coverage, and transcriptomic complexity. Bin-size statistics further quantified changes in molecular and gene detection associated with spatial aggregation. Collectively, these technical assessments, together with the multi-resolution sensitivity analyses, support the quality and reuse potential of the adult Holstein liver Stereo-seq resource and provide a dataset-specific rationale for selecting Bin50 for the primary downstream analyses.

Cross-resolution Leiden clustering and UMAP analyses showed that major transcriptional organization remained evident across Bin50–Bin200, although finer aggregation preserved greater transcriptional granularity (Figure 3). These results support the robustness of the spatial transcriptional structure across aggregation levels.

The bin-size analysis is particularly important because Stereo-seq permits flexible aggregation of the underlying DNB capture array, and there is no universally optimal bin size. Human liver cancer investigators used Bin50 at approximately 25 × 25 µm, bovine placenta investigators selected Bin100 after comparing multiple resolutions, and bovine testis investigators used Bin50 for annotation and downstream analysis. In the present study, Bin50 was selected as a practical intermediate level based on the balance between molecular sensitivity and spatial resolution observed in our dataset. With approximately 0.5 µm DNB spacing, Bin50 represents 50 × 50 DNB positions and an approximate nominal footprint of 25 × 25 µm.

Complementary reference-guided annotation, clustering, marker-gene analysis, and spatial localization supported the biological interpretability of the dataset. Canonical hepatic genes, including *ALB*, *APOA2*, *ORM1*, *CYP1A2*, *ASS1*, and *GLUL*, demonstrated liver-specific transcriptional profiles and both broadly distributed and locally restricted expression patterns. Because Bin50 represents aggregated spatial capture positions, cell-type assignments are interpreted as dominant cell-type identities associated with spatial bins rather than definitive single-cell identities. Detailed quantitative analyses of hepatic zonation and ruminant-specific metabolism will be addressed in future work.

This resource provides an initial reference for bovine hepatic cell-type organization and spatial gene-expression architecture and can support comparative studies across cattle and other mammals. It also provides a foundation for future studies of livestock health, metabolism, and production-related traits, as well as computational method development and benchmarking. A separate Holstein liver Stereo-seq dataset has already been generated and is being analyzed in an independent comparative study, providing an opportunity for future evaluation of reproducibility across biological samples.

Because the current dataset was generated from one healthy adult Holstein cow, it provides an initial reference resource for bovine liver spatial transcriptomics. The extensive spatial coverage and multiple spatial bins enable comprehensive characterization of within-sample transcriptional organization and provide a strong foundation for future studies incorporating additional biological samples. The publicly available raw and processed data further enhance the value and reuse potential of this resource for bovine functional genomics, comparative hepatology, and spatial transcriptomics research.

## 4. Materials and Methods

### 4.1. Tissue Collection and Sample Handling

Liver tissue was collected from a healthy Holstein cow (sample ID: 4815) at the Beltsville Agricultural Research Center (BARC) USDA facility. Tissue was dissected post-mortem promptly, washed in Dulbecco’s Phosphate-Buffered Saline (DPBS) to remove residual blood, and embedded in optimal temperature compound (OCT) (Figure 1a). To preserve RNA quality and tissue morphology, embedding and freezing were performed immediately with care to minimize handling time; the sample collection procedure was completed within minutes of excision.

### 4.2. Stereo-Seq On-Chip Processing and Imaging

Cryosections of 10 µm thickness were prepared on a Leica CM1950 cryostat (Leica Biosystems, Wetzlar, Germany) and stored at −80 °C until processing (Mirxes, Singapore). Sections were placed onto Stereo-seq chips and fixed in methanol at −20 °C for 30 min. Nucleic acid staining and imaging were performed (Qubit ssDNA assay kit; Nikon Eclipse Ti-7 microscope, Nikon Corporation, Tokyo, Japan) to capture tissue morphology and for later spatial alignment (Figure 1a). Following imaging, tissue permeabilization was carried out (37 °C for 5 min) to release mRNA for hybridization to spatially barcoded DNA nanoballs on the chip. Reverse transcription and second-strand synthesis were performed directly on the chip surface according to Stereo-seq protocols. cDNA was purified using VAHTS DNA Clean Beads (Vazyme, Vazyme Biotech Co., Ltd., Nanjing, China) prior to library construction.

### 4.3. Library Construction and Sequencing

Stereo-seq libraries were prepared using the Stereo-seq Library Prep Kit (Cat. No.: 111KL114) following manufacturer instructions [11,12,13,17]. Total RNA integrity was evaluated using the Agilent 4200 TapeStation (Agilent Technologies, Santa Clara, CA, USA); samples with RNA Integrity Number (RIN) ≥ 8.0 were selected for library construction. Library concentration and fragment profiles were assessed by Qubit 4.0 Fluorometer (Thermo Fisher Scientific, Waltham, MA, USA) and Agilent Bioanalyzer (Agilent Technologies, Santa Clara, CA, USA). DNBs were loaded onto patterned nanoarrays and sequenced on a DNBSEQ-T7 (MGI Tech Co., Ltd., Shenzhen, Guangdong, China) instrument with paired-end reads (Read 1: 50 bp; Read 2: 100 bp) following the manufacturer’s run configuration (Figure 1a).

### 4.4. Raw Data Structure and Reading Composition

Read 1 encodes the spatial Cellular Identifier (CID) and Molecular Identifier (MID) within defined base ranges; Read 2 contains the cDNA sequence. CID sequences are used to recover spatial coordinates for each read by aligning them to the chip design map. MID sequences (UMIs) enable PCR duplication correction. During initial processing, reads containing ambiguous nucleotides (e.g., “N”) in MID or CID fields, or reads with low base quality (average quality score < 10), were filtered out.

### 4.5. Primary Processing Pipeline (SAW)

Raw FASTQ files were processed using the published Stereo-seq Analysis Workflow (SAW, v7.0.0) [18] (Figure 1b). Key SAW steps include: CID to coordinate mapping (allowing one-base mismatch to tolerate sequencing/PCR errors), MID filtering, read trimming, and alignment of cDNA reads to the cattle reference genome (ARS-UCD2.0, v2.0) using the STAR (v2.7.2b) aligner [19]. Only uniquely mapped reads with MAPQ > 10 were retained for downstream quantification. UMIs were collapsed to remove PCR duplicates and to construct gene × spatial-bin expression matrices (tissue.gef files). Secondary files produced by SAW include spatial, tissue.raw.gef, tissue.gef, and QC metric tables that were used in downstream filtering.

### 4.6. Bin-Size Sensitivity Analysis

To evaluate how spatial aggregation influenced molecular sensitivity and downstream transcriptional structure, expression data were assessed at Bin20, Bin50, Bin100, Bin150, and Bin200. For each aggregation level, MID counts and the number of detected gene types were examined using scatterplots, violin distributions, and density distributions. Spatial quality metrics were also evaluated at the principal downstream resolutions. Unsupervised clustering and UMAP visualization were compared across the aggregation levels using corresponding preprocessing workflows. Because cross-resolution clustering was assessed primarily through visualization rather than a formal concordance statistic, these comparisons were interpreted as measures of qualitative robustness and resolution-dependent transcriptional granularity rather than evidence of statistical equivalence between clusters.

### 4.7. Downstream Processing (Stereopy, Seurat)

The processed gene expression matrix was imported into Stereopy (v1.6.0) [20], a Python (v3.8.19) toolkit for Stereo-seq data handling and visualization. For downstream single-cell-style analyses, tissue.gef matrices were aggregated into spatial bins (50 µm bin size used for the current analyses) and converted to .h5ad format for compatibility with Anndata, which is the input for further downstream analysis (Figure 1b) as like as Seurat v5.0.0 workflow.

### 4.8. Clustering and Dimensionality Reduction

Principal component analysis was followed by neighborhood graph construction and Leiden clustering to identify transcriptionally distinct spatial domains using the Stereopy pipeline. UMAP was used to visualize relationships among spatial bins in reduced-dimensional space. The primary Bin50 dataset was additionally converted into a Seurat-compatible format for complementary processing and visualization. Seurat workflows were also used after conversion of the processed data into Seurat-compatible objects. Shared nearest-neighbor graph construction used k = 30, and clustering resolution values between 0.45 and 0.75 were tested to identify biologically meaningful transcriptional domains. UMAP visualization was used to represent transcriptomic relationships among spatial bins.

### 4.9. Reference-Guided Cell-Type Annotation

Biological identities were assigned using reference-based annotation approaches, including SingleR (v2.6.0) [21] and Seurat-based label transfer. Cattle-specific single-cell references were used within a contemporary multi-tissue cattle cell atlas that includes bovine liver and hepatocyte subtypes, providing a species-specific reference framework [22]. Annotation confidence was assessed based on concordance among unsupervised clustering, reference-based predictions, cluster-marker expressions, and spatial expression patterns rather than relying on a single annotation algorithm. Seurat’s FindTransferAnchors and IntegrateData functions were also used to support reference-based integration and cell-type prediction [22]. The computational workflow and reference resources used for the analysis are specified in the repository.

### 4.10. Differential Expression and Marker-Gene Analysis

Differential expression analysis between clusters was performed using Seurat FindMarkers. Genes were considered cluster-associated using an adjusted *p*-value ≤ 0.05, absolute log2 fold change ≥ 1, and minimum expression fraction ≥ 0.6 in at least one comparison group. Highly expressed genes across the full dataset were identified and visualized using Scanpy-based plotting. Canonical hepatic marker genes, including *ALB*, *APOA2*, *ORM1*, *GLUL*, and *CYP1A2*, were visualized across spatial coordinates to assess tissue identity, hepatocyte abundance, and spatially variable hepatic expression patterns.

## Data Availability

All raw and processed data generated in this study are publicly available. Raw sequencing data (paired-end Stereo-seq FASTQ files) have been deposited in the NCBI SRA under accession SRX31668717 and are also accessible via GEO under accession GSE315416. Processed datasets, including tissue-level expression matrices (tissue.gef, tissue.raw.gef), annotated h5ad objects, clustering results, spatial coordinates, quality-control metrics, and visualization outputs, are available in the Zenodo repository (https://zenodo.org/records/18112160?preview=1, accessed on 1 September 2026). All analysis scripts and notebooks, including SAW, Stereopy, Seurat, SingleR workflows, and visualization code, are hosted on GitHub (https://github.com/sakter1606/SData_adultLiver4815/, accessed on 1 September 2026). Processing and analysis relied on open-source and publicly available tools, including SAW v7.0.0, Stereopy v1.6.0, Seurat v5.0.0, SingleR v2.6.0, STAR v2.7.2b, FastQC v0.12.0, and standard R v4.4.1/Python 3.8.19 libraries. Exact commands, software versions, and parameter files are provided in the GitHub repository to ensure full reproducibility. Comprehensive README and metadata files accompany the repository, detailing sample information, processing workflows, and instructions required to fully reproduce the analyses. All resources are provided in accordance with FAIR data principles to support reuse and benchmarking.

## Supplementary Materials

The following supporting information can be downloaded at: https://www.mdpi.com/article/10.3390/ijms27177844/s1.
