# Supplementary material for: A High-Resolution Stereo-Seq Spatial Transcriptomic Resource for Adult Holstein Cattle Liver
Source: Int J Mol Sci. 2026 Sep 2;27(17):7844. doi: 10.3390/ijms27177844 (PMC13565877; doi:10.3390/ijms27177844)
Supplement: Supplementary file 1 [file ijms-27-07844-s001.zip › ijms-4443609-supplementary.pdf]

Supplemental Materials

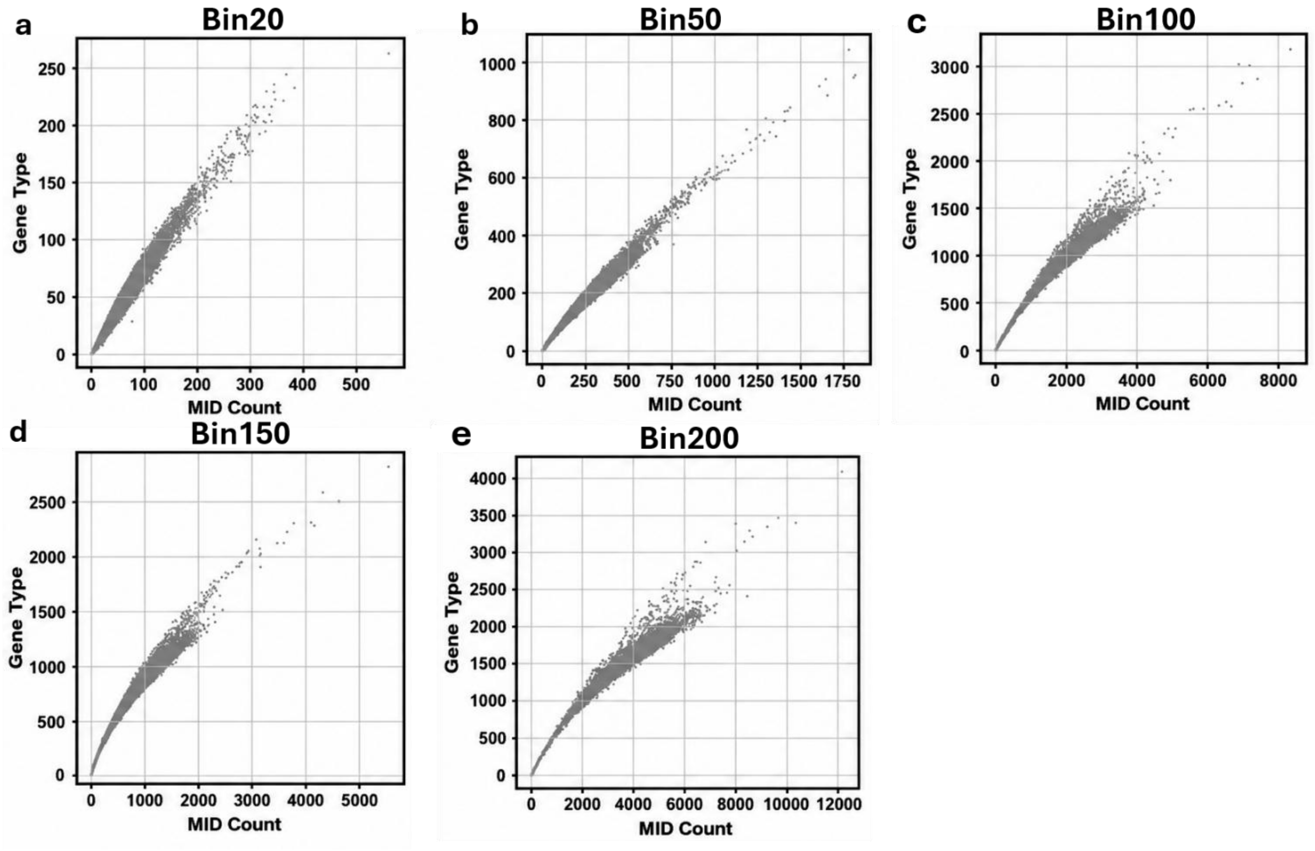

Figure S1. Relationship between molecular recovery and detected-gene complexity across Stereo-seq bin sizes. Scatterplots show MID counts versus the number of detected gene types for (a) Bin20, (b) Bin50, (c) Bin100, (d) Bin150, and (e) Bin200. A positive relationship between molecular counts and detected-gene complexity is observed across all aggregation levels, with both measures showing broader ranges as increasing numbers of DNB capture positions are combined.

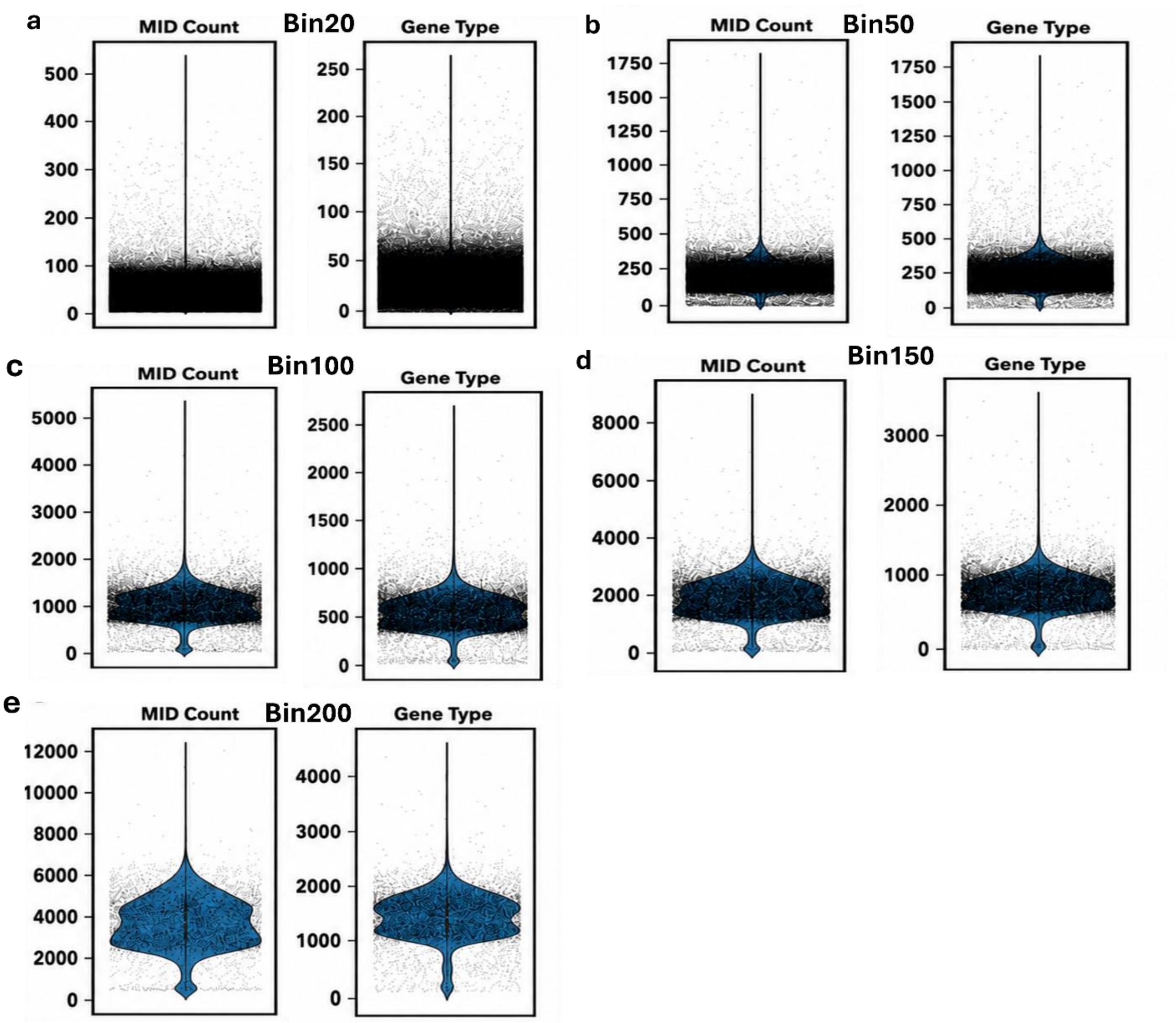

Figure S2. Bin-size sensitivity of molecular and gene detection. Violin plots show MID counts and detected-gene numbers at Bin20, Bin50, Bin100, Bin150, and Bin200. Increasing aggregation increases molecular and gene recovery, whereas smaller bins retain finer spatial granularity. Bin50 provides a practical intermediate balance and was selected for the primary analysis.

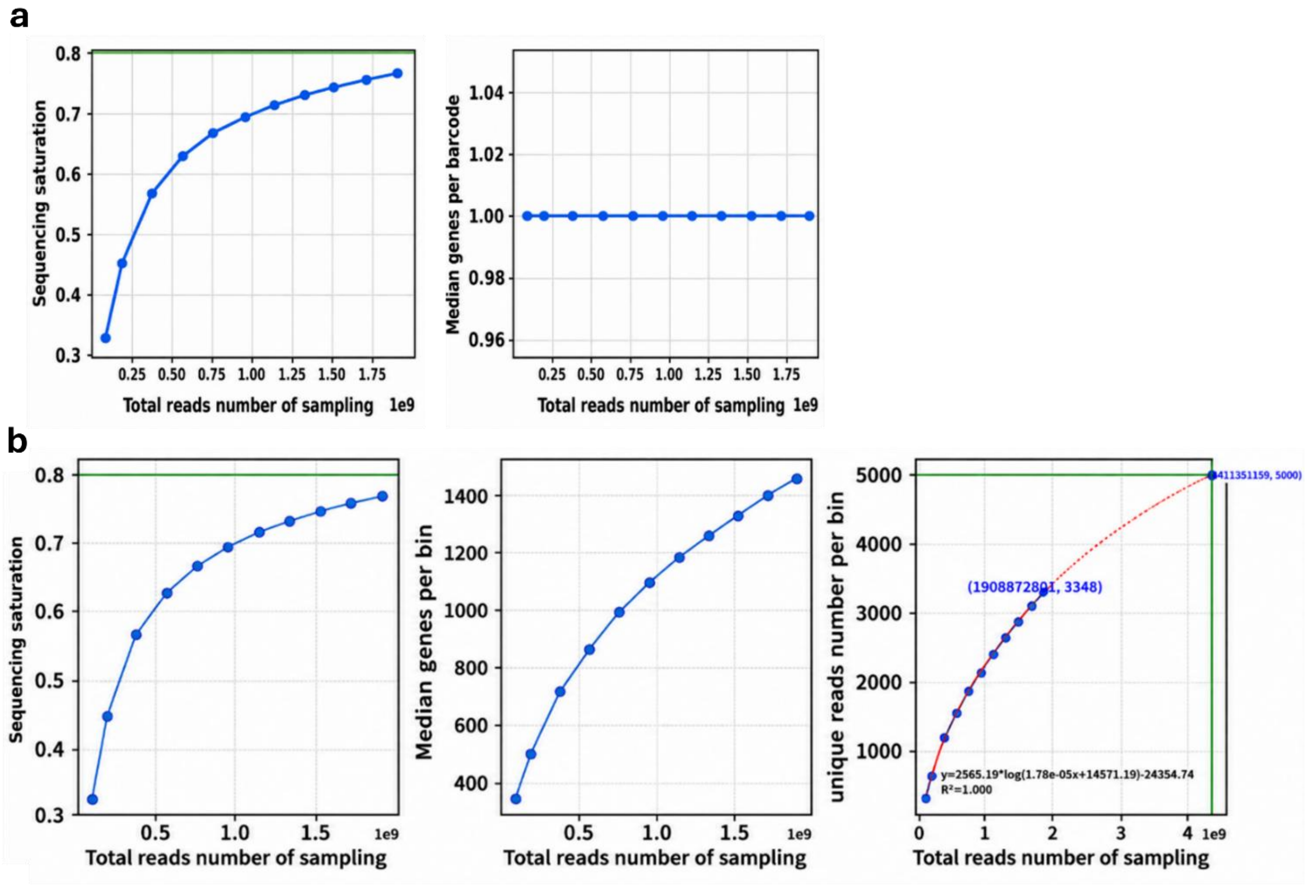

FigureS3 Sequence saturation plot. (a) Saturation plot 1x1 and (b) Saturation plot 200x200.

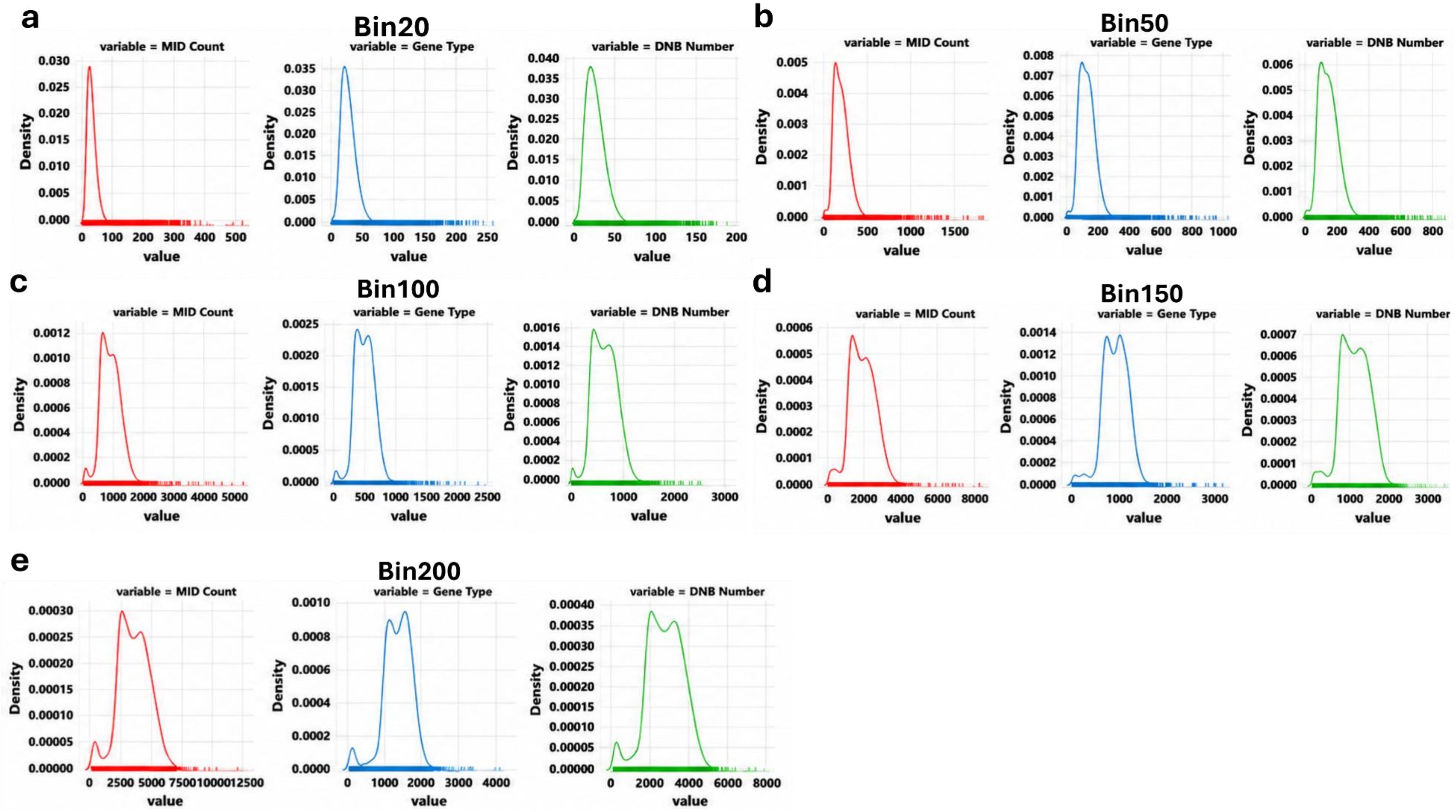

Figure S4. Density distributions of Stereo-seq quality metrics across spatial aggregation levels. Kernel-density distributions summarize MID counts, detected gene types, and DNB-related coverage metrics for Bin20, Bin50, Bin100, Bin150, and Bin200. Progressive changes in the distributions with increasing bin size reflect increased molecular aggregation and transcriptomic complexity. These results complement the scatterplot and violin-plot analyses by illustrating the overall distributional effects of spatial aggregation.

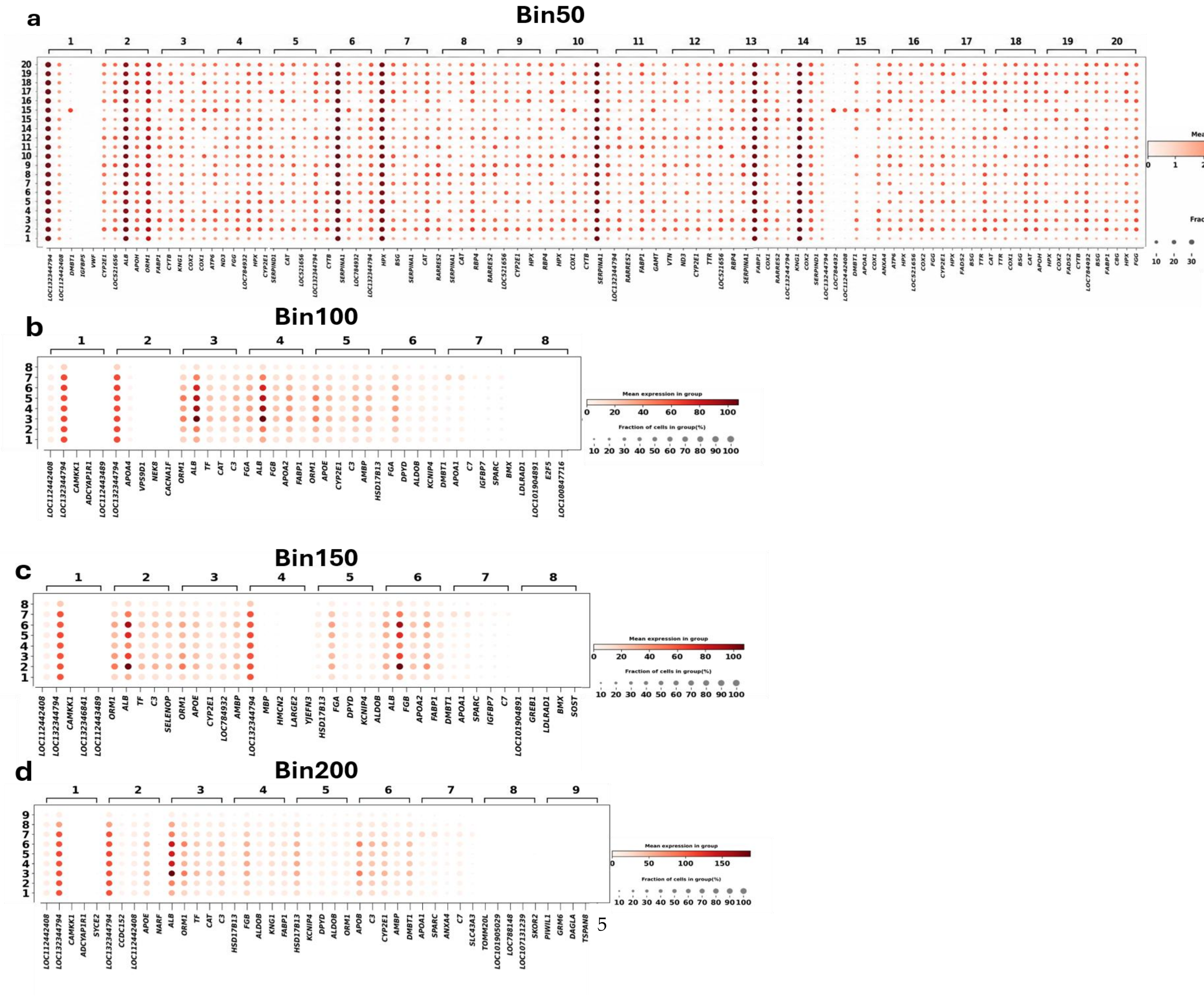

a
Bin50
b
Bin100
c
Bin150
d
Bin200
Mean expression in group
Fraction of cells in group(%)

Figure S5. Cluster-marker expression across of Leiden clusters Stereo-seq aggregation levels. Dot plots summarize cluster-associated marker-gene expression for (a) Bin50, (b) Bin100, (c) Bin150, and (d) Bin200. Dot size represents the fraction of spatial bins expressing each marker, while color represents the corresponding group-level mean expression based on the plotting workflow. Cluster-associated transcriptional signatures remain detectable across aggregation levels, although cluster numbers, boundaries, and marker distributions vary with spatial resolution.

Table S1. Tissue statistical analysis with stain image.

**Contour_area**	19355668
**Ratio**	6.97%
**Total_gene_type**	18680
**MID_counts: 23697028**	23697028
**Fraction_MID_in_spots_under_tissue: 89.33%**	89.33%
**Reads_under_tissue**	1037631816
**Fraction_reads_in_spots_under_tissue**	82.53%

TableS2. Tissue cut and bin size statistics.

Bin Size	1	20	50	100	150	200
Mean_reads_per_spot	5.87	1493.47	9270.03	36692.66	81954.97	144035.52
Median_reads_per_spot	3	1357.00	8729.00	35356.00	79663.00	141362.00
Mean_gene_type_per_spot	1.08	28.48	151.55	484.52	910.56	1379.81
Median_gene_type_per_spot	1	27	146	482	919	1408
Mean_Umi_per_spot	1.22	34.22	212.47	840.53	1876.84	3297.21
Median_Umi_per_spot	1	31	201	814	1843	3258

Table S3. Up to 20 differentially expressed genes (DEGs) used for cell-type assignment in adult cattle liver.

P value	Avg log_2FC	Pct.1	Pct.2	P value adjusted	Cluster	Gene
9.66321720027842e-36	6.873577	0.125	0.002	1.80508897301201e-31	B cells	EHF
1.09982049223761e-36	5.014498	0.292	0.012	2.05446467949986e-32	B cells	C7
4.3453326977188e-11	4.75705	0.125	0.008	8.11708147933871e-07	B cells	NFKB1
5.63767537256439e-10	4.415056	0.125	0.009	1.05311775959503e-05	B cells	LYN
2.3083538509676e-10	4.176291	0.125	0.008	4.31200499360748e-06	B cells	MPEG1
3.24711809005795e-06	4.002007	0.125	0.014	0.060656	B cells	VPS41
2.94984821599704e-07	3.970514	0.125	0.012	0.00551	B cells	NCOR2
1.12824213866616e-30	3.774506	0.5	0.04	2.10755631502838e-26	B cells	BOLA-DRA
6.85376138905241e-05	3.768288	0.125	0.018	1	B cells	TNRC6C
1.72934706576649e-12	3.607007	0.25	0.025	3.23042031885181e-08	B cells	BLA-DQB
4.80077229722599e-07	3.343237	0.167	0.021	0.008968	B cells	PUM2
0.000364	3.302843	0.125	0.021	1	B cells	VAV3
1.79787970524084e-05	3.257846	0.125	0.016	0.335844	B cells	MRPL54
1.75796222564702e-05	3.151938	0.125	0.016	0.328387	B cells	ITPR1
4.89627399912704e-05	3.145055	0.125	0.017	0.914624	B cells	BRI3
8.32701844959302e-07	3.061937	0.167	0.021	0.015555	B cells	BOLA-DRB3
2.1380615174753e-13	3.046886	0.458	0.073	3.99389891464387e-09	B cells	CD74
9.84971871450179e-05	3.035347	0.125	0.018	1	B cells	NT5C
0.002206	2.961941	0.125	0.026	1	B cells	BICC1
0.001407	2.951095	0.125	0.025	1	B cells	CST3
3.01122324598484e-172	2.609756	0.317	0.118	5.62496502349969e-168	Cholangiocytes	DMBT1
2.47163316764411e-186	2.244007	0.359	0.137	4.6170107571592e-182	Cholangiocytes	APOA1
3.09002991279509e-87	1.642556	0.261	0.116	5.77217587710123e-83	Cholangiocytes	ANXA4
2.12331306176133e-55	1.172636	0.191	0.087	3.96634879937016e-51	Cholangiocytes	ACTG1
4.89619686997919e-76	1.000222	0.343	0.177	9.14609575312113e-72	Cholangiocytes	RPS12
0	3.013502	0.185	0.024	0	Hepatocytes	AKR1C3
0	2.772685	0.25	0.037	0	Hepatocytes	F2
6.0399058176388e-228	2.568031	0.126	0.023	1.12825440673493e-223	Hepatocytes	ACSM1
0	2.434154	0.39	0.076	0	Hepatocytes	AHSG
1.78820205284379e-288	2.364516	0.161	0.031	3.3403614347122e-284	Hepatocytes	ACSM2B
2.88257495943028e-262	2.312673	0.153	0.032	5.38465002421577e-258	Hepatocytes	ADH4
0	1.752547	0.328	0.099	0	Hepatocytes	ALDH1A1
1.06645919017336e-137	1.564609	0.122	0.042	1.99214576724384e-133	Hepatocytes	AHCY
0	1.509687	0.321	0.118	0	Hepatocytes	APOC3
4.84657625433528e-102	1.405615	0.102	0.039	9.05340444309831e-98	Hepatocytes	ALDH2
0	1.273802	0.652	0.331	0	Hepatocytes	APOH
2.80347342395185e-102	1.145565	0.135	0.063	5.23688835594205e-98	Hepatocytes	APOB
5.22016065741634e-121	1.102904	0.159	0.075	9.75126010805373e-117	Hepatocytes	A1BG
3.64414619177386e-203	1.070866	0.264	0.132	6.80726508623356e-199	Hepatocytes	MSMO1

7.90755961117839e-152	1.06339	0.221	0.115	1.47713213536812e-147	Hepatocytes	C4BPA
1.0100182180227e-23	3.354676	0.114	0.025	1.88671403126641e-19	Kupffer cells	CST3
3.73370832455334e-87	3.196093	0.263	0.039	6.97456715026564e-83	Kupffer cells	BOLA-DRA
5.94046685982365e-91	3.1377	0.209	0.025	1.10967920941506e-86	Kupffer cells	BLA-DQB
1.90140924241254e-27	3.091179	0.111	0.021	3.55183246482663e-23	Kupffer cells	BOLA-DRB3
3.99045385414906e-59	2.642439	0.313	0.073	7.45416779955044e-55	Kupffer cells	CD74
4.60274634044495e-44	2.37236	0.306	0.086	8.59793016395116e-40	Kupffer cells	TMSB10
1.53314552986825e-39	2.291147	0.253	0.065	2.8639158497939e-35	Kupffer cells	TMSB4X
1.06505118767493e-35	2.053168	0.222	0.056	1.98951561857677e-31	Kupffer cells	CFD
9.48764697184642e-18	2.048406	0.141	0.042	1.77229245434091e-13	Kupffer cells	RPL35A
2.13261903580273e-07	1.959946	0.212	0.121	0.003984	Kupffer cells	DMBT1
2.02583722077949e-12	1.869707	0.101	0.031	3.78426392841608e-08	Kupffer cells	RPS23
3.40773547753331e-12	1.856621	0.114	0.038	6.36564987203222e-08	Kupffer cells	RPS29
1.16816962481172e-13	1.807091	0.141	0.049	2.18214085914828e-09	Kupffer cells	RPL39
1.64411742682476e-07	1.758041	0.125	0.056	0.003071	Kupffer cells	RPLP0
3.88099381409784e-13	1.678128	0.199	0.085	7.24969644473477e-09	Kupffer cells	BOLA
3.88072325642039e-15	1.642449	0.189	0.072	7.24919104299329e-11	Kupffer cells	JSP.1
7.34768776042776e-07	1.460122	0.128	0.061	0.013725	Kupffer cells	RPS3A
3.88079505340897e-13	1.446325	0.296	0.153	7.24932515976795e-09	Kupffer cells	B2M
1.4767801986953e-07	1.44624	0.192	0.103	0.002759	Kupffer cells	LOC783680
4.3889284249007e-18	1.394746	0.401	0.208	8.19851829771451e-14	Kupffer cells	ACTB
5.05569050154192e-263	4.832011	0.1	0.005	9.4440298568803e-259	NK T cells	KRT19
3.58752840930262e-174	4.573751	0.125	0.012	6.70150306857729e-170	NK T cells	PKHD1
0	4.530034	0.159	0.009	0	NK T cells	MGP
0	4.510507	0.181	0.011	0	NK T cells	C7
2.06842751165247e-186	4.120911	0.1	0.007	3.86382259176682e-182	NK T cells	LOC506707
9.03645690024035e-165	4.041395	0.116	0.011	1.6880101489649e-160	NK T cells	SLC4A4
1.86005341183233e-219	3.912604	0.462	0.119	3.4745797733028e-215	NK T cells	DMBT1
1.62847627622872e-175	3.843125	0.125	0.011	3.04199368399524e-171	NK T cells	GNG11
3.60239798382513e-149	3.590818	0.434	0.139	6.72927943378534e-145	NK T cells	APOA1
3.26449637055127e-129	3.4842	0.126	0.015	6.09807922018976e-125	NK T cells	PIGR
5.2781961462717e-81	3.257058	0.109	0.017	9.85967040123554e-77	NK T cells	PLD1
3.95343004504551e-90	3.19069	0.138	0.024	7.38500732414502e-86	NK T cells	CST3
2.76983164263996e-80	3.07815	0.104	0.016	5.17404550845144e-76	NK T cells	VIM
1.28656450443309e-69	3.032716	0.104	0.018	2.40330249428101e-65	NK T cells	PRKCE
1.96078468102521e-206	3.008548	0.388	0.084	3.66274578415509e-202	NK T cells	TMSB10
1.27777007399594e-157	3.006656	0.235	0.041	2.38687449822441e-153	NK T cells	RPL35A
5.57095649209423e-94	2.977248	0.126	0.02	1.0406546727232e-89	NK T cells	COL3A1
1.32296473092707e-104	2.877085	0.146	0.024	2.47129811737177e-100	NK T cells	PTMA
2.79298031413524e-98	2.865307	0.162	0.03	5.21728722680463e-94	NK T cells	RPS23
7.15632505682238e-190	2.860344	0.324	0.064	1.33680152061442e-185	NK T cells	TMSB4X
5.09568063112641e-211	4.856719	0.103	0.005	9.51873141894413e-207	T cells	KRT19
5.07994722227093e-226	4.460114	0.147	0.01	9.4893414112021e-222	T cells	MGP

6.98456484913653e-191	4.45337	0.115	0.007	1.3047167138187e-186	T cells	LOC506707
1.17683121918899e-145	4.260126	0.131	0.012	2.19832071744504e-141	T cells	PKHD1
4.22669341046177e-174	4.230794	0.142	0.012	7.89546329074259e-170	T cells	GNG11
8.69926462236556e-173	4.185871	0.135	0.011	1.62502263145789e-168	T cells	SLC4A4
2.02789170173711e-215	4.185185	0.154	0.011	3.78810169884491e-211	T cells	C7
1.60917164002414e-152	4.06567	0.447	0.12	3.00593262356509e-148	T cells	DMBT1
2.56165316146777e-101	3.680315	0.415	0.139	4.78516810562179e-97	T cells	APOA1
3.22388516651295e-126	3.645822	0.142	0.015	6.02221749104618e-122	T cells	PIGR
1.57598896474125e-82	3.420184	0.124	0.017	2.94394738613665e-78	T cells	PLD1
6.81005855311096e-54	3.407697	0.105	0.018	1.27211893772113e-49	T cells	PRKCE
9.71854869524846e-83	3.203484	0.135	0.02	1.81542489627241e-78	T cells	COL3A1
1.66375115558224e-72	3.052832	0.11	0.015	3.10788715862763e-68	T cells	RPL26
7.55644390472324e-124	3.018384	0.202	0.03	1.4115437214023e-119	T cells	RPS23
1.74825213244666e-157	2.961222	0.392	0.085	3.26573498341036e-153	T cells	TMSB10
1.91615987782775e-112	2.903966	0.23	0.041	3.57938665178224e-108	T cells	RPL35A
5.73590630377717e-104	2.852067	0.204	0.035	1.07146729754557e-99	T cells	RPS7
2.5099298495736e-56	2.772767	0.115	0.02	4.68854895900348e-52	T cells	RPS4X
2.94076551178551e-67	2.744816	0.335	0.117	5.49334997601532e-63	T cells	ANXA4
